# An Unexpected Cause of De Novo Urge Incontinence Following Artificial Urinary Sphincter Placement: A Case Report

**DOI:** 10.3390/jcm15176622

**Published:** 2026-08-27

**Authors:** Ante Jakšić, Iva Žuža, Ivan Marin Sušanj, Ivan Vukelić, Josip Španjol, Dean Markić, Argimiro Collado Serra, Antun Gršković

**Affiliations:** 1 Department of Urology, Clinical Hospital Center Rijeka, 51000 Rijeka, Croatia; ante.jaksic@medri.uniri.hr (A.J.); ramm.ivanmarin@gmail.com (I.M.S.); vukelic.ivan@yahoo.com (I.V.); josip.spanjol@medri.uniri.hr (J.Š.); dean.markic@medri.uniri.hr (D.M.); 2 Department of Urology, Faculty of Medicine, University of Rijeka, 51000 Rijeka, Croatia; 3 Department of Radiology, Clinical Hospital Center Rijeka, 51000 Rijeka, Croatia; iva.zuza276@gmail.com; 4 Department of Radiology, Faculty of Medicine, University of Rijeka, 51000 Rijeka, Croatia; 5 Functional Urology Unit, Department of Urology, Fundación IVO, 46009 Valencia, Spain; argicollado@gmail.com

**Keywords:** urinary incontinence, urge, urinary sphincter, artificial, prostatectomy, fracture fixation, internal, case report

## Abstract

**Background:** Stress urinary incontinence is one of the most common and debilitating complications following radical prostatectomy. Artificial urinary sphincter (AUS) implantation is the gold standard treatment for moderate to severe post-prostatectomy incontinence. Common complications include device infection, mechanical failure, component migration, urethral erosion, and urethral atrophy, which often require device removal and subsequent reimplantation. To our knowledge, erosion or migration of orthopedic osteosynthesis screws into the lower urinary tract causing new-onset urinary symptoms after AUS implantation has not previously been reported. **Case Presentation:** A 57-year-old man with an implanted AUS presented to the emergency department with fever, dysuria, and de novo urge incontinence. Intravenous antibiotic therapy was initiated, resulting in resolution of the fever, after which further diagnostic evaluation was performed. Cystoscopy demonstrated normal urethral coaptation and intact urethral mucosa at the cuff site. However, three orthopedic osteosynthesis screws from previous pelvic fracture fixation were identified protruding immediately below the bladder neck. The screws were removed by open surgery during ongoing empirical antimicrobial therapy. Four weeks later, the AUS was reactivated. At 8-month follow-up, the patient remained asymptomatic and maintained the same continence status as following AUS implantation. **Conclusions**: A detailed history of previous pelvic surgery and orthopedic fixation, together with review of available imaging, is important in candidates for AUS implantation. In patients presenting with new-onset urinary symptoms after AUS implantation, alternative etiologies should be considered, with cross-sectional imaging and cystoscopy used selectively according to the clinical presentation.

## 1. Introduction

Prostate cancer is the second most prevalent cancer among men, with nearly 1.5 million new cases diagnosed worldwide each year [1,2]. One of the primary treatment options for localized disease is radical prostatectomy, which is performed in approximately 20–30% of patients [3]. Despite excellent oncological outcomes, stress urinary incontinence (SUI) remains one of the most common functional complications following surgery, with a reported prevalence ranging from 4% to 40%, depending on the definition of continence and the timing of assessment [4]. Because SUI can significantly impair quality of life, a substantial proportion of affected patients require surgical treatment. Current surgical options for post-prostatectomy SUI include male sling implantation and the artificial urinary sphincter (AUS), which remains the gold standard for moderate to severe incontinence. AUS implantation is associated with high continence rates, with approximately 60% of patients achieving complete continence and up to 90% requiring no more than one safety pad per day [5]. However, the procedure is associated with several potential complications. Early complications include hematoma formation, urinary retention, urinary tract infection, component migration, and fluid loss, whereas late complications include device infection, urethral atrophy, mechanical failure, and urethral erosion, the most devastating complication of AUS implantation [6]. Among these, urethral erosion is not only the most devastating complication but also the most common cause of foreign bodies identified within the urinary tract of patients with an AUS. To our knowledge, the presence of unrelated foreign bodies within the lower urinary tract causing new-onset urinary symptoms after AUS implantation has not previously been reported. Here, we present a case of delayed erosion of orthopedic osteosynthesis screws into the proximal urethra, presenting with de novo urge urinary incontinence in a patient with a functioning artificial urinary sphincter.

## 2. Case Presentation

A 57-year-old man underwent open radical prostatectomy without pelvic lymph node dissection in May 2022 for clinically favorable intermediate-risk prostate cancer. However, final histopathological examination demonstrated prostate adenocarcinoma, pT3aNx, with a Gleason score of 4 + 5 and a positive surgical margin. Postoperatively, PSA remained detectable at 1.2 ng/mL. Following multidisciplinary team discussion, the patient received salvage whole-pelvic radiotherapy to a total dose of 63 Gy, initiated four months after surgery, in combination with androgen deprivation therapy using the LHRH agonist goserelin, which was continued for three years. Following radiotherapy, PSA decreased to 0.2 ng/mL and remained stable during the documented follow-up period. After treatment, the patient reported mild stress urinary incontinence requiring no more than two pads per day.

In May 2023, he sustained polytrauma in a motorcycle accident, including a multifragmentary pelvic fracture, bilateral radial fractures, multiple rib fractures, and a left scapular fracture. The pelvic fracture was initially stabilized with an external fixator, followed by definitive internal fixation one month later. By October 2023, he had regained full mobility without orthopedic aids. However, his urinary incontinence had markedly worsened, most likely because of additional sphincter dysfunction associated with the pelvic trauma.

Following orthopedic recovery, he sought surgical treatment for urinary incontinence. Preoperative evaluation demonstrated a 24-h pad weight of 1100 g and an International Consultation on Incontinence Questionnaire–Short Form (ICIQ-SF) score of 21. The patient did not report urinary urgency or urge incontinence and had no post-void residual urine. Urethrocystoscopy revealed no abnormalities. Urodynamic evaluation, including uroflowmetry, cystometry, and pressure-flow studies, was attempted but could not be completed because of continuous urinary leakage. Urine culture was sterile. The patient’s blood glucose level was within the normal range at the time of AUS implantation, with a perioperative HbA1c of 6.4%. In September 2024, an AMS 800 (InhibiZone) artificial urinary sphincter was implanted. The patient received intravenous antibiotic prophylaxis with amoxicillin/clavulanic acid 1.2 g three times daily and amikacin 0.5 g/day, starting one day before surgery. Amikacin was discontinued after three days, while amoxicillin/clavulanic acid was continued for seven days. Intraoperatively, the bulbar urethral circumference measured 5.5 cm. Given the patient’s history of diabetes mellitus, previous pelvic radiotherapy, and extensive periurethral fibrosis encountered intraoperatively, a 6.0-cm cuff was intentionally selected to minimize the risk of future urethral complications. The postoperative course was uneventful, with a marked improvement in continence and quality of life. The 24-h pad weight decreased to 80 g, allowing the patient to resume recreational motorcycle riding.

Fourteen months after AUS implantation, the hemodynamically stable patient presented to the emergency department with fever, malaise, dysuria, de novo urinary urgency, and urge urinary incontinence. Physical examination, including abdominal, genital, perineal, and digital rectal examination, was unremarkable. Laboratory evaluation revealed a serum creatinine level of 123 μmol/L, leukocyte count of 10.0 × 10^9^/L, and C-reactive protein level of 12.3 mg/L. Blood and urine samples for culture were obtained before initiation of antimicrobial therapy and remained sterile. The AUS appeared to function normally on clinical examination.

**Figure 1:**
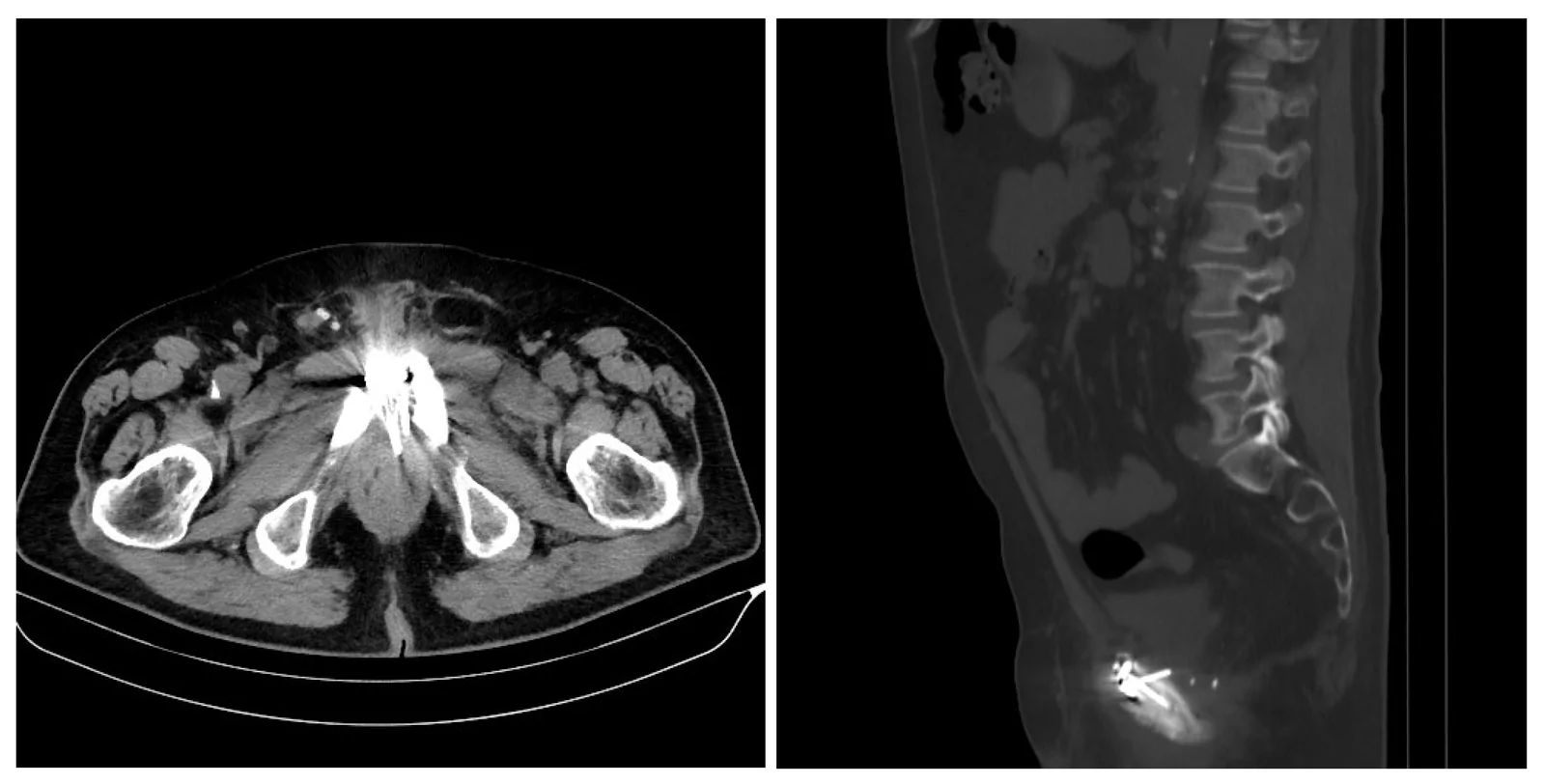
Contrast-enhanced computed tomography (axial and sagittal planes) demonstrating orthopedic osteosynthesis screws from previous pelvic fracture fixation projecting beyond the pubic bone into the adjacent pelvic soft tissues.

Renal ultrasonography was unremarkable, with no evidence of post-void residual urine. However, contrast-enhanced computed tomography demonstrated several orthopedic osteosynthesis screws from the previous pelvic fixation projecting beyond the pubic bone into the adjacent pelvic soft tissues (Figure 1). Given the patient’s febrile presentation, the presence of implanted prosthetic and orthopedic material, and the initial suspicion of urethral cuff erosion, empirical broad-spectrum intravenous therapy with piperacillin–tazobactam (4.5 g every 8 h) was initiated following consultation with an infectious disease specialist and continued for 14 days.

**Figure 2:**
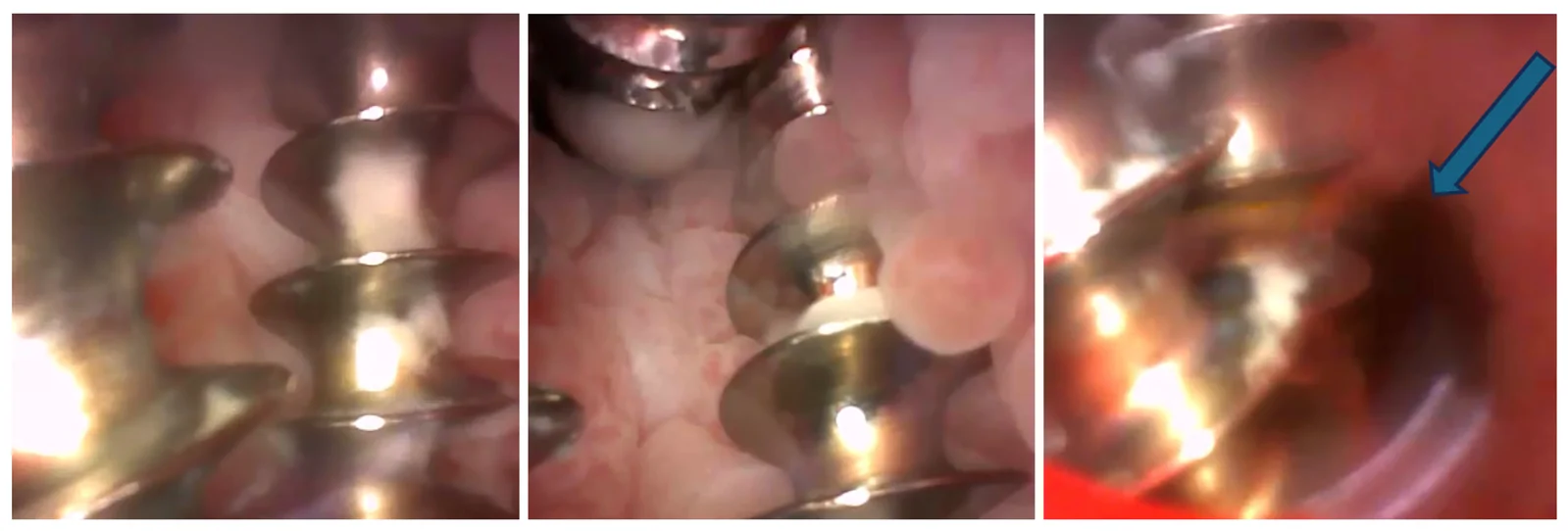
Cystoscopic view of three orthopedic osteosynthesis screws protruding into the right ventrolateral aspect of the proximal urethra immediately below the bladder neck. Blue arrow indicates the bladder neck opening.

Cystoscopy was performed after clinical stabilization. Following insertion of a flexible cystoscope into the penile urethra, good urethral coaptation was observed. The AUS was then deflated and deactivated and, although urethral cuff erosion was initially suspected, cystoscopy demonstrated intact urethral mucosa at the cuff site. Unexpectedly, three osteosynthesis screws were found protruding into the right ventrolateral aspect of the proximal urethra, immediately below the bladder neck (Figure 2). The AUS remained deactivated during subsequent catheterization with a 12-Fr transurethral catheter.

**Figure 3:**
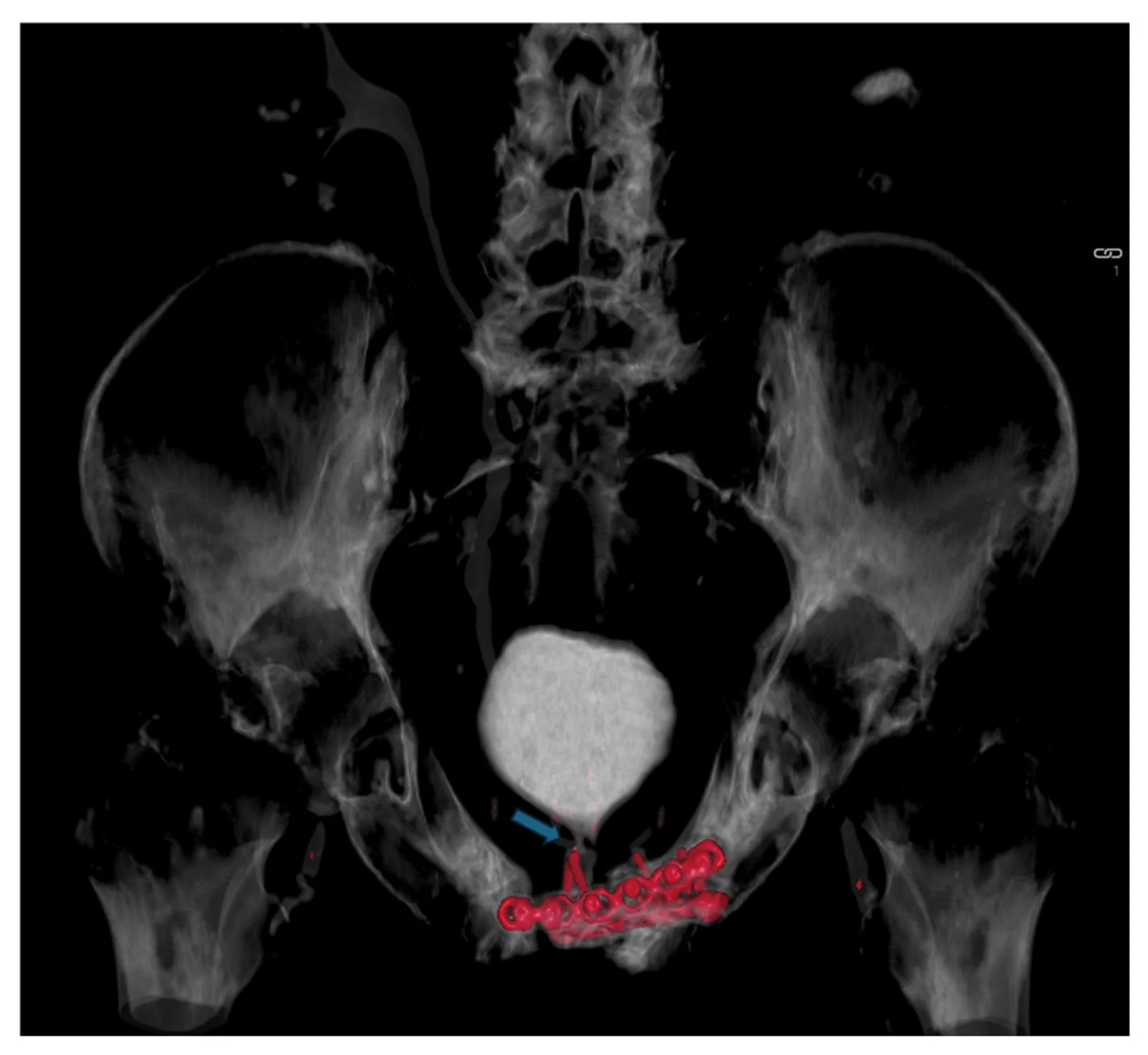
Excretory-phase CT of the abdomen and pelvis with volume-rendered technique (VRT), bone, and metal reconstructions demonstrating orthopedic osteosynthesis screws from previous pelvic fracture fixation (highlighted in red) projecting beyond the pubic bone into the adjacent pelvic soft tissues (frontal plane). The screw tips are surrounded by contrast-opacified urine at the level of the urethra (arrow), demonstrating their close relationship to the urethral lumen.

**Figure 4:**
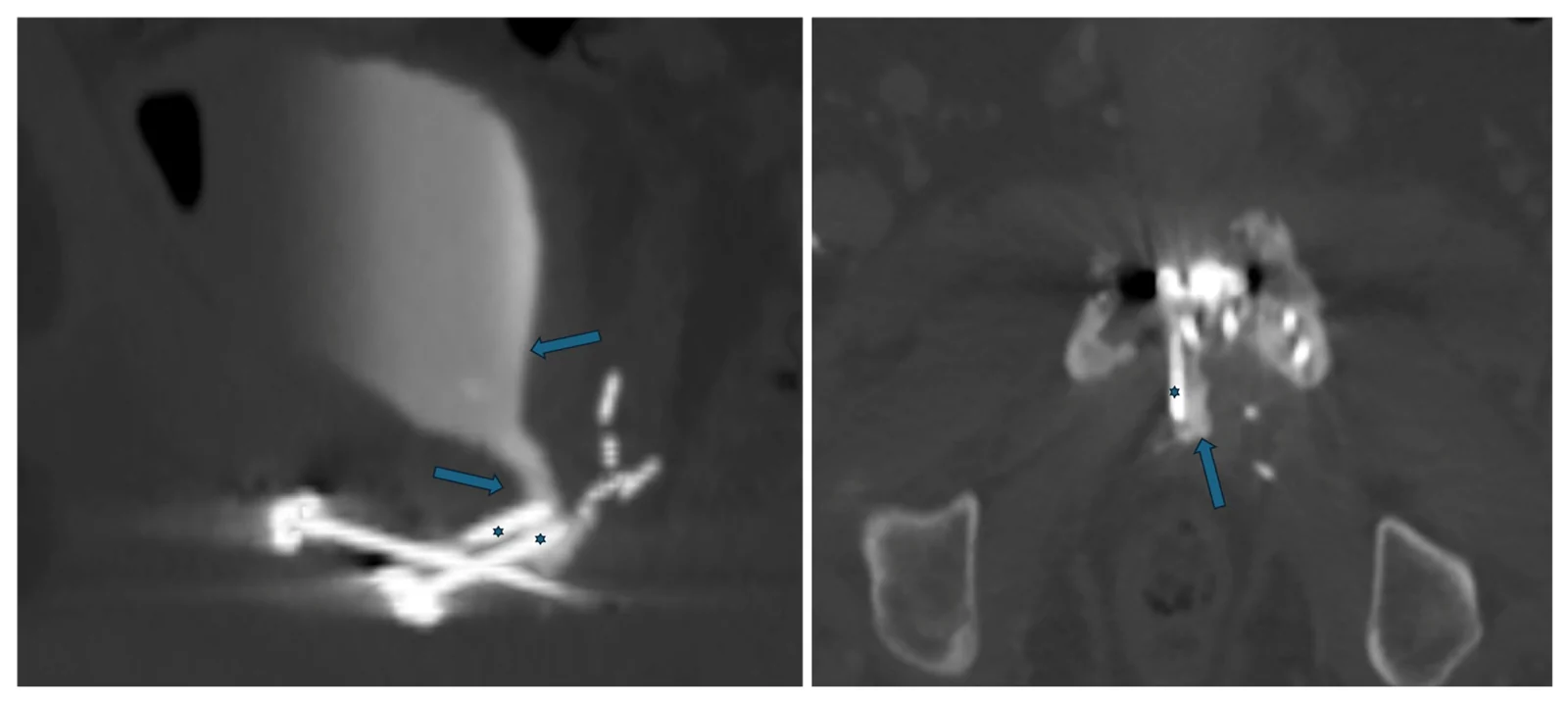
Maximum intensity projection (MIP) images with a 3-mm slice thickness in the sagittal (**left**) and axial (**right**) planes, demonstrating the relationship between the osteosynthesis screws and the urethra. The bladder and urethral lumen (blue arrows) are delineated by contrast-opacified urine, allowing for assessment of their relationship to the adjacent screw tips (blue asterisks).

**Figure 5:**
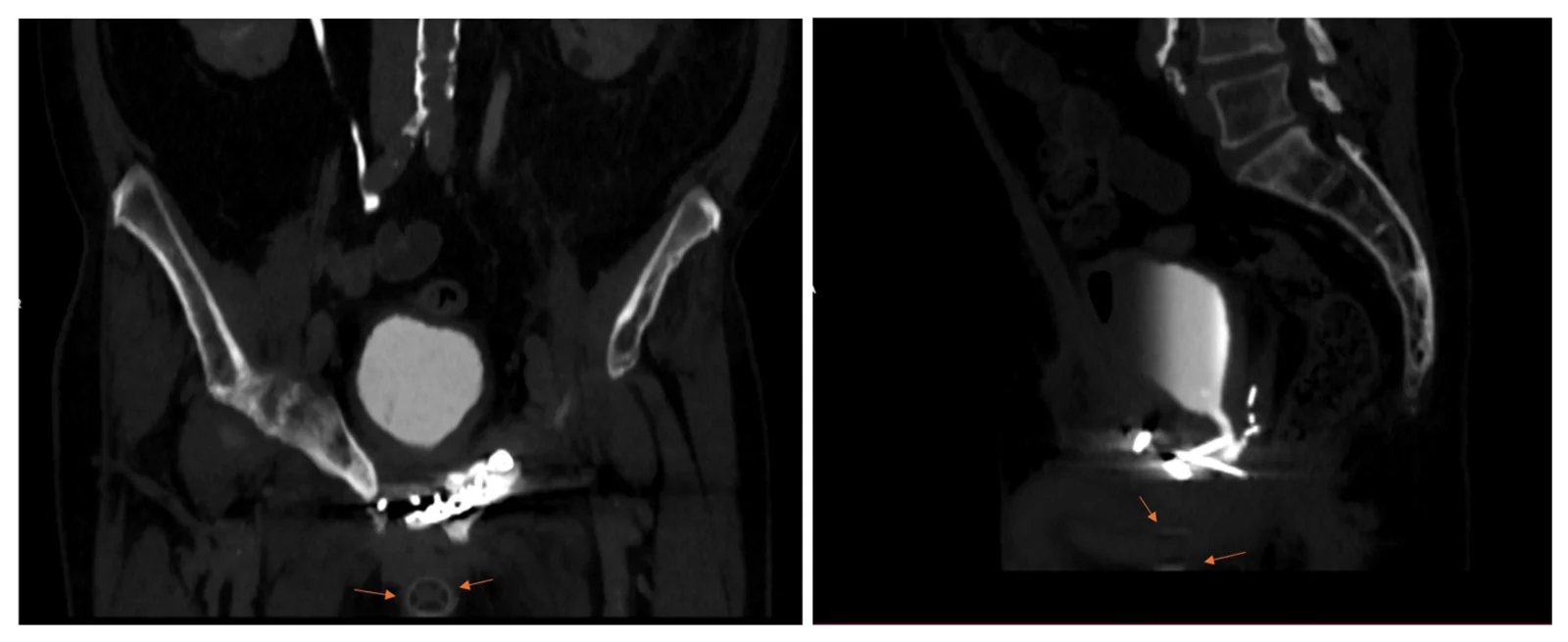
Maximum intensity projection (MIP) images with a 3-mm slice thickness in the frontal (**left**) and sagittal (**right**) planes, demonstrating the AUS cuff (arrows) surrounding the bulbar urethra.

Additional CT image reconstructions demonstrated the exact position of the screws relative to the urethra in the frontal, sagittal, and axial planes, as well as the position of the sphincter cuff around the bulbar urethra (Figure 3, Figure 4 and Figure 5).

Following consultation with the orthopedic team, pelvic ring stability was confirmed clinically and radiologically, and removal of the orthopedic fixation hardware was planned. All screws and fixation plates were removed through the previous Pfannenstiel incision by the orthopedic team, with a urologist present throughout the procedure. The patient remained on intravenous piperacillin–tazobactam (4.5 g every 8 h) during the procedure as part of the previously initiated 14-day antimicrobial regimen. No additional intraoperative urethral imaging or endoscopic assessment was performed. Hardware removal was uneventful, with no clinically evident urinary leakage or significant bleeding. Conservative management with continued transurethral catheter drainage was selected.

The patient was discharged two days after hardware removal with the AUS deactivated and a 12-Fr transurethral catheter in place. The catheter was maintained for three weeks after hardware removal. No urethrographic or endoscopic evaluation was performed before catheter removal. Suprapubic and perineal ultrasonography demonstrated no pelvic or periurethral fluid collections. In the absence of clinical signs of urinary leakage or infection, the catheter was removed, while the AUS remained deactivated for an additional week before reactivation.

At the 8-month follow-up, the patient remained asymptomatic and maintained the same continence status as after AUS implantation. The 24-h pad weight was 50–80 g over two pads, the ICIQ-SF score was 10, and no post-void residual urine was detected. Urgency and urge urinary incontinence had completely resolved.

The key events in the patient’s clinical course are summarized in Table 1.

## 3. Discussion

This case describes a previously unreported cause of de novo lower urinary tract symptoms (LUTS) in a patient with a functioning AUS. Although delayed migration or erosion of orthopedic fixation screws into the lower urinary tract has been described, to our knowledge, this is the first reported case occurring in a patient with an implanted AUS. The coexistence of these two conditions created a unique diagnostic challenge, as urethral cuff erosion would ordinarily be considered the most likely cause of the patient’s sudden onset of urgency, dysuria, and urge urinary incontinence.

Previous studies have identified pelvic irradiation and small cuff size as factors associated with an increased risk of urethral erosion [7,8]. In the present case, given the history of pelvic radiotherapy and extensive periurethral fibrosis encountered intraoperatively, a 6.0-cm cuff was selected despite a measured bulbar urethral circumference of 5.5 cm to avoid excessive urethral compression. This represented an individualized intraoperative decision rather than a standardized cuff-sizing strategy. Importantly, cystoscopy at the time of the acute presentation demonstrated intact urethral mucosa at the cuff site, with no evidence of cuff erosion.

The delayed nature of the screw erosion is supported by the patient’s clinical course. Preoperative urethrocystoscopy performed before AUS implantation demonstrated no evidence of urethral injury or screw protrusion, indicating that erosion occurred after implantation. Previously reported cases of orthopedic screw migration, protrusion or erosion have involved predominantly the bladder and only rarely the urethra. Patients typically present with gross hematuria, dysuria, recurrent urinary tract infection, urinary retention, or other nonspecific LUTS. The interval between pelvic fracture fixation and symptom onset varies considerably, ranging from several days to almost two decades, highlighting that hardware-related complications may occur long after fracture healing [9,10,11,12,13].

While the exact mechanism by which the screws eroded into the urethra cannot be established, chronic micromotion of the pelvic fixation hardware may have contributed to gradual erosion through the surrounding periurethral soft tissues and eventual exposure within the proximal urethra. Restoration of physical activity following successful AUS implantation may have further contributed to this process, although this remains speculative. Repeated mechanical contact between the protruding screw tips and the adjacent urethral wall may also have contributed to progressive erosion and eventual intraluminal exposure immediately below the bladder neck.

The mechanism responsible for the patient’s de novo urgency and urge urinary incontinence is likely multifactorial. Chronic irritation of the bladder neck and proximal urethra by the protruding screws may have induced local inflammation and stimulation of bladder afferent pathways, a well-recognized mechanism underlying urgency and other storage LUTS, despite a normally functioning AUS [14]. The resolution of these symptoms following screw removal may support this hypothesis.

However, this mechanism remains hypothetical, as antibiotic therapy, AUS deactivation, and catheter drainage were performed concurrently with hardware removal. Therefore, we cannot determine with certainty whether screw removal alone contributed to the resolution of the lower urinary tract symptoms.

The present case also highlights several important diagnostic and therapeutic considerations. In patients with an AUS presenting with sudden-onset LUTS, cuff erosion should remain the primary differential diagnosis. However, clinicians should also consider alternative etiologies, particularly in patients with a history of pelvic trauma or orthopedic fixation. Cross-sectional imaging combined with cystoscopy proved essential for establishing the correct diagnosis and planning appropriate treatment.

After identification of the screws, the AUS remained deactivated, and urinary drainage was established to minimize urinary extravasation and facilitate urethral healing before definitive treatment. Given the normal function of the AUS, intact urethral mucosa at the cuff site, and the distinct anatomical locations of the screws in the retropubic region and the cuff around the bulbar urethra, preservation of the AUS was considered appropriate. The choice of urinary drainage required balancing the need to promote urethral healing against the potential risks associated with prolonged transurethral catheterization in the presence of an AUS cuff. Although suprapubic diversion would have avoided urethral instrumentation, a small-caliber 12-Fr transurethral catheter was considered an acceptable alternative. Keeping the AUS deactivated for an additional week after catheter removal was intended to further minimize mechanical stress on the healing urethra. Nevertheless, suprapubic diversion may represent a reasonable alternative in similar cases, particularly when prolonged urinary drainage is anticipated.

Following AUS reactivation, the patient regained his pre-event level of continence without recurrent LUTS.

This case emphasizes that delayed migration of orthopedic fixation hardware should be considered in the differential diagnosis of new-onset LUTS in patients with a history of pelvic fracture surgery, even years after the initial trauma. A thorough evaluation, including detailed surgical history, cross-sectional imaging, and endoscopic assessment, is essential for accurate diagnosis and may enable preservation of a functioning artificial urinary sphincter.

## 4. Conclusions

Although cuff erosion remains the most likely cause of new-onset urinary symptoms after AUS implantation, alternative etiologies should be considered, particularly in patients with a history of pelvic trauma and orthopedic fixation. A thorough evaluation should include a detailed history of previous pelvic surgery and review of existing imaging, with selective use of cross-sectional imaging and cystoscopy according to the clinical presentation. Early recognition and multidisciplinary management may enable successful treatment while preserving a functioning artificial urinary sphincter.

## Figures and Tables

**Table 1:** Timeline of the patient’s clinical course according to the CARE guidelines.

Date/Period	Clinical Event and Management
May 2022	Open radical prostatectomy without pelvic lymph node dissection. Final histopathology demonstrated prostate adenocarcinoma, pT3aNx, Gleason score 4 + 5, with a positive surgical margin. Postoperative PSA remained detectable at 1.2 ng/mL.
September 2022	Salvage whole-pelvic radiotherapy (63 Gy) was initiated four months after surgery in combination with androgen deprivation therapy using goserelin.
May 2023	Motorcycle accident resulting in polytrauma, including a multifragmentary pelvic fracture. The pelvic fracture was initially stabilized with an external fixator.
June 2023	Definitive internal fixation of the pelvic fracture with osteosynthesis screws and plates.
October 2023	Full mobility regained without orthopedic aids. Urinary incontinence had markedly worsened following pelvic trauma.
September 2024	AMS 800 (InhibiZone) artificial urinary sphincter implanted for severe SUI (24-h pad weight, 1100 g; ICIQ-SF score, 21). Intraoperative bulbar urethral circumference was 5.5 cm, and a 6.0-cm cuff was implanted.
Post-AUS implantation	Marked improvement in continence, with a decrease in 24-h pad weight to 80 g. The patient resumed recreational motorcycle riding.
November 2025	Presentation with fever, malaise, dysuria, de novo urinary urgency, and urge urinary incontinence. CT demonstrated osteosynthesis screws projecting beyond the pubic bone into the adjacent pelvic soft tissues. Blood and urine cultures obtained before antimicrobial therapy remained sterile. Empirical intravenous piperacillin–tazobactam was initiated.
During the same hospitalization	Cystoscopy demonstrated good urethral coaptation and intact mucosa at the cuff site. The AUS was deflated and deactivated. Three osteosynthesis screws were found protruding into the right ventrolateral proximal urethra immediately below the bladder neck. A 12-Fr transurethral catheter was placed for drainage. All orthopedic screws and fixation plates were removed by the orthopedic team. The AUS was preserved and remained deactivated. Conservative management with transurethral catheter drainage was continued.
3 weeks after hardware removal	In the absence of clinical signs of urinary leakage or infection and with no pelvic or periurethral fluid collections on ultrasonography, the transurethral catheter was removed.
4 weeks after hardware removal	AUS was reactivated after an additional week following catheter removal.
8-month follow-up	The patient remained asymptomatic. Urgency and urge urinary incontinence had completely resolved. The 24-h pad weight was 50–80 g over two pads, ICIQ-SF score was 10, and no post-void residual urine was detected.

## Data Availability

No new datasets were generated or analyzed in this study. Data supporting the findings of this case report are available from the corresponding author upon reasonable request, subject to patient confidentiality and institutional regulations.

## Abbreviations

The following abbreviations are used in this manuscript:

AUS	Artificial Urinary Sphincter
ICIQ-SF	International Consultation on Incontinence Questionnaire–Short Form
LUTS	Lower Urinary Tract Symptoms
MIP	Maximum Intensity Projections
PSA	Prostate-Specific Antigen
SUI	Stress Urinary Incontinence
VRT	Volume-Rendered Technique
